# Does Acupuncture Improve Quality of Life for Patients with Pain Associated with the Spine? A Systematic Review

**DOI:** 10.1155/2011/301767

**Published:** 2010-09-29

**Authors:** Shao-chen Lu, Zhen Zheng, Charlie Changli Xue

**Affiliations:** Traditional & Complementary Medicine Research Program, Health Innovations Research Institute and Discipline of Chinese Medicine, School of Health Sciences, RMIT University, Bundoora, VIC 3083, Australia

## Abstract

This paper aimed to evaluate the effectiveness of acupuncture for qualities of life (QoL) in patients suffering from pain associated with the spine (PAWS). Acupuncture has been shown to reduce pain severity, but its effect on QoL is unknown. PubMed, CINAHL, and Cochrane Central Register of Controlled Trials as well as EMBASE were searched. Published randomized controlled trials on PAWS comparing acupuncture with waiting-list or sham interventions were considered. Eight out of 186 trials were included. For physical functioning, acupuncture was better than waiting-list at immediate and short-term followups; and was better than sham interventions at immediate assessment (SMD = 0.40. 95% CI 0.06 to 0.74). For mental functioning, acupuncture was better than waiting-list at short-term followup and sham interventions at intermediate-term followup (SMD = 0.27. 95% CI 0.03 to 0.51). A similar effect was observed on pain reduction. Discrepancies in point selection for relieving anxiety and insufficient training of trial acupuncturists were also identified. Acupuncture has a moderate effect on the improvement of physical functioning and pain for PAWS patients in the short term; but the effect for mental functioning is small and delayed. Future trials should address point selection and consistency in the qualifications of trial acupuncturists.

## 1. Introduction

Pain associated with the spine (PAWS) is defined as pain along the spine, pain on both sides of the spine, and discogenic sciatica [1]. Epidemiological data show a high correlation between neck and low back pain [2] and these pains can be considered as one condition. PAWS has a life-time prevalence of between 54% to 80% [3] and considerably impacts on patients' quality of life (QoL), including sleep [4], mood [4], psychological well-being [1], and functional ability [5]. Assessing QoL represents three of the six core domains in the IMMPACT (initiative on methods, measurement, and pain assessment in clinical trials) recommendations [6]. 

QoL consists of physical, mental, social and role functioning components [7]. Trials have suggested that pain and QoL are strongly related [8]. Existing systematic reviews (SRs) of acupuncture for neck or low back pain (LBP) have focused on pain, function, or disabilities assessment [9, 10] but assessment of other aspects of QoL, such as mental and social functioning, is lacking. 

Acupuncture is a holistic therapy as it deals with the main symptoms and the general wellbeing of the patients concurrently. There is evidence that acupuncture improves disability and function [11–13] as well as mental functioning [14–16]. 

The present paper evaluates the effectiveness of acupuncture on QoL and pain for patients with PAWS. The specific aims of the paper are to assess (1) both the physical and mental effects of acupuncture on PAWS when compared with waiting-list or sham intervention (SI); (2) the correlation between reduction of pain and improvement in QoL when practicable. The physical functioning consists of physical ability, disability, working status, and daily activity functioning of the patients. The mental functioning consists of mental ability, spirituality, and emotion.

## 2. Methods

### 2.1. Inclusion and Exclusion Criteria

Only randomised controlled trials (RCTs) were included. All patients with PAWS due to arthritis, disc protrusion, trauma, degeneration, or nonspecific origin were considered. The pain duration included both acute (less than three months) and chronic (over three months). Trials were excluded if they included PAWS due to cancer, tumour, infection, metastatic diseases, fractures, or neurological origin conditions.

Trials must have used acupuncture involving skin penetrations in the treatment group. Acupuncture was defined as “needle insertion on the body, including the use of the filiform needle, electroacupuncture, warming needle (needling with moxibustion on top of the needles), three edge needle, dermal needle (tapping with the so-called seven-star needle or plum blossom needle), and intradermal needle” [17]. The control groups were waiting-list or SI. SI included sham acupuncture, sham TENS, and sham laser treatment.

To be included, the trial must have satisfied three criteria: (1) have included at least one QoL measure, using a validated questionnaire, such as Short Form 36 Health Survey Questionnaire (SF-36), General Health Questionnaire, Roland Morris Questionnaire (RMQ), European Quality of Life, Nottingham Health Profile, Hospital Anxiety Depression Scale, Pain Disability Index, Northwick Park Neck Pain Questionnaire, Oswestry Disability Index, Neck Disability Index, Neck Pain and Disability Index, or Japanese Orthopaedic Association Assessment; (2) have used the visual analogue scale (VAS) for pain assessment. Using the VAS rather than other pain measurements was to simplify the pain assessment; and (3) received a score of at least three on the Jadad scale [18].

The durations of the followup period [9] were defined as

- immediate followup: up to seven days after the last treatment;
- short-term followup: between seven days and three months after the last treatment;
- intermediate-term followup: between three months and one year after the last treatment;
- long-term followup: more than one year after the last treatment.

### 2.2. Search Strategy for Identification of Trials

The following databases were searched from their inception to October 24, 2008: PubMed, Cumulative Index to Nursing and Allied Health Literature (CINAHL) (via EBSCO) and the Cochrane Central Register of Controlled Trials. EMBASE (via Science Direct) was searched from its inception to October 11, 2006. Search terms and subject headings included randomised controlled trials, controlled trials, neck pain, back pain, low back pain, ankylosing spondylosis, disc protrusion, acupuncture, and acupressure were used and adapted for different databases as necessary. References lists from the included trials were searched to find other potential papers.

Languages of publication were limited to English, German, and Chinese. Authors of the trials were contacted if more information was needed.

One author (LS) conducted citation identification and trial selection. The procedure was double checked by another author (ZZ).

### 2.3. Methodological Assessment

The reporting quality of the trials was assessed with modified Jadad Scale [18] by one author (LS) and double checked by TS. Any disagreement was resolved by discussion with another author (ZZ).

### 2.4. Data Extraction

One author (LS) extracted data for demographics, treatment procedure, outcomes (Table 1) and items of Standards for Reporting Interventions in Controlled Trials of Acupuncture (STRICTA) [27], and data were double checked by TS. For trials reporting more than one QoL assessment, the assessment measuring the broader aspects of QoL was chosen for meta-analysis. For instance, when a trial reported both the Neck Disability Index and SF-36 physical functioning, only the SF-36 data were used. When trials only reported the combined value of physical and mental functioning (e.g., the total value of SF-36), the data were not included in the meta-analysis.

### 2.5. Data Analysis

The results of trials with similar control interventions and duration of followup were pooled together. Data were analysed using RevMan 4.3. Random-effects model (REM), standard mean difference (SMD), and 95% confidence interval (95% CI) were used for outcomes reported in a continuous data format. SMD was used because different measures were frequently used and REM was used to incorporate heterogeneity among the trials. Relative risk and 95% CI were used for outcomes reported in dichotomous data format. The effect size was categorised as small, medium, and large if it was equal or more than 0.20, 0.50, and 0.80, respectively [28].

### 2.6. Sensitivity Analysis [29]

When sufficient data were available, trials were grouped into (1) neck pain (NP), (2) LBP, and (3) other spinal pain including thoracic sacral and coccygeal pain, in order to explore the heterogeneity if there was any. Otherwise, the statistical method was changed from REM to fixed effect model (FEM) to see if there was any change in the results.

## 3. Results

### 3.1. Description of Trials

**Figure 1:**
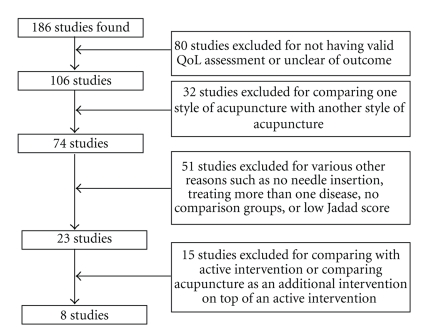
A flowchart of study selection.

Of the 1,090 citations found and screened, 186 clinical trials were identified. Only eight trials met the selection criteria [19–26]. The main reason for exclusion was not having any QoL data or the types of outcome assessment were unclear. The selection process is illustrated in Figure 1. 

Table 1 provides details of the demographic data. Overall, all included trials appropriately randomised participants. Three trials did not blind participants from the type of interventions, of which one compared acupuncture with waiting-list [26] and two [22, 24] compared acupuncture with sham TENS. Acupuncturists were not blinded in any of the trials. Assessors were blinded in five trials [20–24] and assessor blinding was not reported in three trials [19, 25, 26]. One trial had a very large number of participants (*n* = 2841) [26], while the other trials had 26 to 301 participants. Three trials were multicentre studies [19, 20, 26], four were single centred [21, 22, 24, 25], and the remaining one trial did not provide the number of centres involved but mentioned that three acupuncturists delivered treatments [23]. The duration of pain ranged from less than 12 weeks [23] to an average of 15.8 years [19].

Five of the included trials were on LBP [19, 21–23, 26] and the remaining three were on NP [20, 24, 25]. The most frequently used measurements were VAS for pain [19–26] and SF-36 for QoL [19, 20, 22, 24–26]. The other commonly used measurements were Hannover Functional Ability Questionnaire (validated German questionnaire Funktionsfragebogen Hannover-Rucken) [19, 26], and RMQ [21, 23].

Of the six trials compared acupuncture with SI, three compared it with sham acupuncture [20, 21, 23] and the other three with sham TENS [22, 24, 25]. One of the remaining two trials compared acupuncture with waiting-list [26] and the other with both sham acupuncture and waiting-list [19]. Seven trials reported the immediate followup [19–25]; five the short-term followup [20, 21, 23, 25, 26]; and three the intermediate-term followup [19, 24, 25]. None assessed long-term followup, that is, more than one year.

The meta-analysis results are summarised in Tables 2 and 3.

### 3.2. Physical Functioning

Two trials compared acupuncture with waiting-list, including 2808 LBP participants [19, 26]. Acupuncture was more effective than waiting-list at the immediate (SMD = 0.68. 95% CI 0.39 to 0.97) [19] and short-term followups (SMD = 0.51. 95% CI 0.43 to 0.59) [26]. 

**Figure 2:**
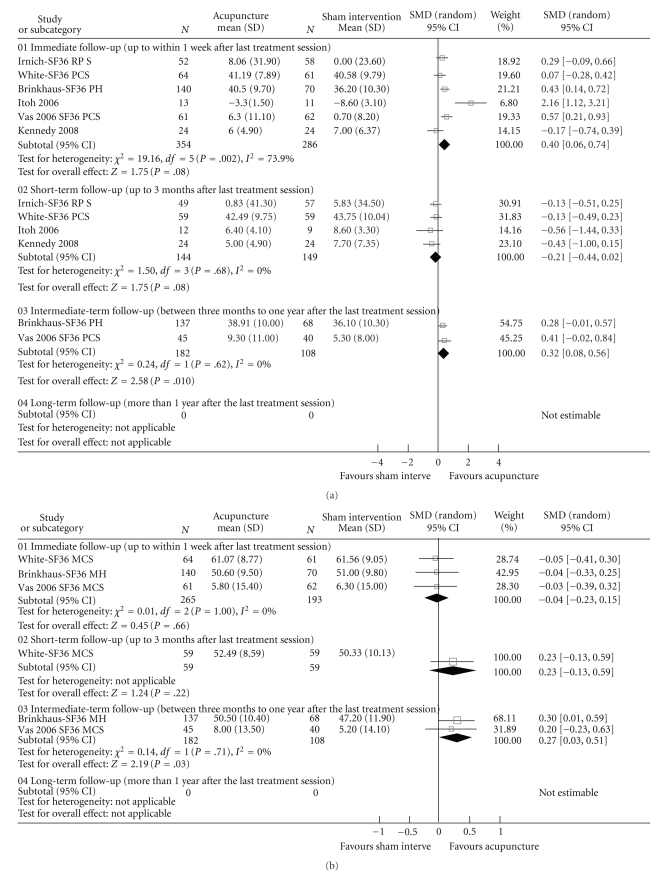

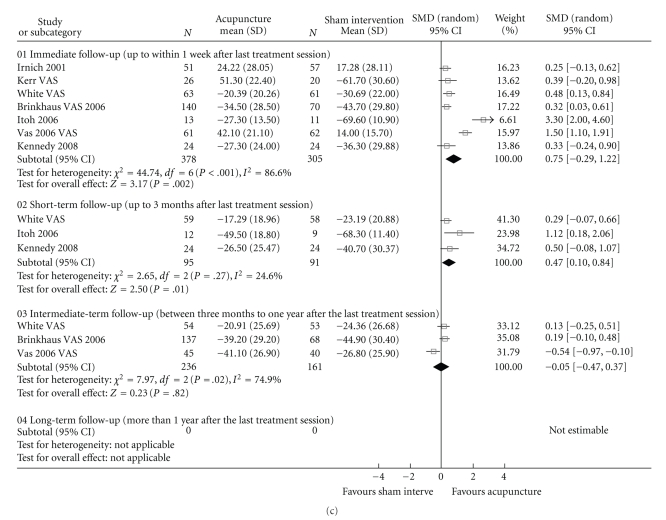
Meta-analyses of the effects of acupuncture on QoL and Pain. (a) Acupuncture versus sham interventions for physical functioning. (b) Acupuncture versus sham interventions for mental functioning. (c) Acupuncture versus sham interventions for pain.

Figure 2(a) illustrates the results of the meta-analysis of trials comparing acupuncture with SI. Three LBP trials [19, 21, 23] and three NP trials [20, 24, 25] totalling 640 participants were included. At the immediate followup, acupuncture was more effective than SI [19–21, 23–25]. Sensitivity test was conducted but this did not change the result. To identify the source of heterogeneity, we analysed the NP trials separately from the LBP trials. The results favoured acupuncture on NP (SMD = 0.31. 95% CI 0.02 to 0.60 I^2^= 48%) but showed no group difference for LBP. Acupuncture was not better than SI at the short-term followup; but had a small superior effect at the intermediate-term followup.

### 3.3. Mental Functioning

Two trials compared acupuncture with waiting-list including 2808 LBP participants [19, 26]. One trial showed no difference between the groups [19] at the immediate followup; and the other showed a small effect favour acupuncture (SMD = 0.23. 95% CI 0.15 to 0.31) [26] at the short-term followup. 

One LBP [19] and two NP trials [24, 25] compared acupuncture with SI totalling 458 participants. As shown in Figure 2(b), there was no difference between the two groups at the immediate and short-term followups but a small effect favouring acupuncture was detected at the intermediate-term followup. (SMD = 0.27. 95% CI 0.03 to 0.51 I^2^ = 0%).

### 3.4. Pain

Two LBP trials compared acupuncture with waiting-list totalling 2808 participants. Acupuncture was better than waiting-list with a large to medium effect at both the immediate (SMD = 0.88. 95% CI 0.58 to 1.17) [19] and short-term followups (SMD = 0.69. 95% CI 0.61 to 0.77) [26].

Three NP [20, 24, 25] and four LBP trials [19, 21–23] compared acupuncture with SI including 683 participants. As shown in Figure 2(c), acupuncture was more effective than SI at the immediate and short-term followups [21, 23, 25]. No group difference was identified at the intermediate-term followup. The sensitivity test and subgroup analysis were conducted and this did neither change the result nor the heterogeneity. 

Due to the small number of trials included, no correlation between QoL and pain could be determined.

### 3.5. STRICTA

The STRICTA data are presented in Table 4. In one trial, the trial physicians were allowed to treat participants similarly to their usual practice for 15 sessions over three months as long as they used needle acupuncture without laser therapy [26]. The paper did not clearly describe what styles of acupuncture the trial physicians used. A methodological paper could not be found for the information needed. Due to lack of details, this paper is not included in the summary that follows, so only seven out of eight trials are described. 

Six trials used both local and distal points [19, 20, 22–25], with two selecting points based on traditional Chinese medicine theory [20, 24]. Three trials allowed auricular points in addition to body acupuncture [19, 20, 24]. Five trials applied manual stimulation to achieve *deqi* sensation (a distending or numb sensation at the acupuncture sites) [19, 22–25], and the remaining two mentioned “local twitch response” without specify manual or electrical stimulation [20, 21]. Needle retention was at least 20 minutes in six out of seven trials [19, 20, 22–25] and 10 minutes in one trial [21]. Acupuncture treatment was given once to twice a week for 5–12 times in five trials [19, 20, 22, 24, 25]. 

Four clinical trials had one acupuncturist conduct all the acupuncture treatments [21, 22, 24, 25] whereas the other four clinical trials involved three [23] to 3486 [26] trial acupuncturists. The training and experiences of acupuncturists in each trial varied. Only one trial employed acupuncturists with at least four years of formal training [21], four trials had medical acupuncturists with at least 140 hours of training [19, 26], or physiotherapy acupuncturist with 80 hours of accredited courses [23, 25], and the remaining trials did not report the training background [20, 22, 24]. In five trials, years of practice varied from three to 15 years [19, 21, 23–25], and two did not report [20, 22]. 

Reporting of other aspects of STRICTA varies as shown in Table 4.

## 4. Discussion

This SR demonstrates that acupuncture may improve physical functioning and pain and it has a delayed, small effect on mental functioning in patients with PAWS.

Our result for pain reduction in PAWS is consistent with those of two former SRs published in 2005 and 2007, where acupuncture was found to be more effective than waiting-list and SI [9, 10]. The current paper found the effect size was between medium to large. Unfortunately, the two previously published SRs did not assess mental functioning, which is important to pain sufferers, nor analysed general QoL outcomes, such as those included in SF-36.

The present paper has the following characteristics: firstly, it updates the current state of evidence on acupuncture for PAWS by including five recently published trials [19, 21, 23, 24, 26]. One of these has a sample size of 2841 participants, which may strengthen the quality of the evidence presented in this paper [26]. Secondly, only high-quality trials are included to ensure clinical relevance of findings from this paper. All trials were assessed based on the Jadad scale as part of the selection process to reduce bias due to the lack of blinding and randomisation as trials with such weaknesses are more likely to produce positive outcomes [30, 31]. Thirdly, to determine the benefit of acupuncture as a stand alone therapy, only trials comparing acupuncture with waiting-list or sham acupuncture are included. That is, trials comparing acupuncture with other intervention or assessing acupuncture as an adjunctive intervention to a standard therapy [32] were excluded. 

As a result, only two [20, 25] out of the ten trials reviewed in Trinh's SR [10] and one [22] out of the 35 trials in Furlan's SR [9] met the selection criteria for this paper. 

The findings of the present SR are, however, contradictory to those of a recently published SR by Madsen [33], which concluded that acupuncture analgesia was clinically irrelevant and could be due to a lack of blinding of acupuncturists or psychological factors. The difference in results might be due to: (1) the present SR only included trials that scored three or higher on the Jadad scale; (2) different endpoints were used to assess the benefits of acupuncture for pain management. Madsen focused on pain reduction only whereas this SR considered both QoL and pain; and (3) this SR aimed to evaluate acupuncture for PAWS while Madsen's review covered acupuncture studies on all pain conditions. Consequently, only one [19] out of the thirteen trials included in Madsen's SR met the inclusion criteria for this paper. 

Similar to SRs by Furlan and Trinh, a high level of heterogeneity was detected in the present review. The sources of heterogeneity could be the variation in the pain history, assessment using different QoL instruments, and/or the use of different acupuncture treatment regimens. Some trials required a minimum of one month of pain duration whereas other trials required more than a six-month history of pain. Because not all trials used SF-36, we extracted data from SF-36 and RMQ. We use SMD instead of Weighted Mean Difference for the data analysis to address variation in pain assessment tools.

As for acupuncture regimen, the methods of point selection varied from one trial to another. Most trials used a combination of distal and local points on the body, and three trials used additional auricular acupuncture [19, 20, 24]. The number of treatments offered also varied from five to 15. Such variations could be major sources of heterogeneity. 

Inclusion of both LBP and NP in one review is unlikely to be a major source of heterogeneity. These are related conditions and have been combined in clinical trials [34, 35]. Furthermore, as shown in the analysis, separating the two types of pain did not change the overall result for pain or mental functioning but there was a significant result for physical functioning in LBP trials only. 

Medications such as opioids and coxibs have been reported to improve QoL, especially symptom distress scores [36]. The adverse effects of opioids ranged from mild ones such as nausea, vomiting, drowsiness, clouding of the mind, constipation, and difficulty in urination [37], to severe ones such as respiratory depression and hypotension [37]. Long-term opioid usage is associated with dependence and addiction [38]. The adverse effects of nonsteroidal anti-inflammatory drugs ranged from mild, such as gastritis [39], to serious, such as aggravation of renal failure [39] and increased risk of cardiovascular events [40]. This limited their use to short term. In contrast, the adverse effects of acupuncture are short-lasting and mild, such as tiredness and drowsiness [41], making acupuncture potentially a safer choice of treatment for improving QoL of chronic PAWS patients.

**Figure 3:**
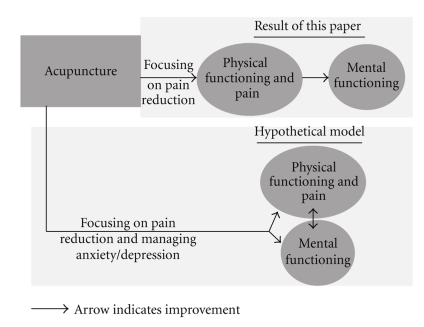
A hypothetical framework of the effects of acupuncture on QoL.

We only found a delayed, small effect supporting acupuncture as improving mental functioning at three months to one year after the end of the treatments. This is consistent with findings from both the controlled and uncontrolled trials [11, 42]. Other interview or survey studies have also reported that after acupuncture, patients reported improved emotional strength [43], became calmer [14, 44, 45], and experienced positive emotional changes [46]. Considering these findings, the point selections in trials could be improved and be more focused on the mental aspects of the treatment. In the included trials in the current SR, generally a formulated protocol or a set of points is applied to address pain; whereas in clinical practice, practitioners choose different points from one session to another to address patients' needs at that session as well as the chief complaint. Furthermore, the traditionally trained acupuncturist focuses on patients' emotional issues as well as their pain. One of the main Chinese medicine principles for treating pain is to “calm the *shen* (mind)” [47], which is consistent with the understanding of modern medicine that pain and anxiety/mental stress reinforce each other [48]. In acupuncture, the commonly used points for calming the mind or “*shen*” include HT3, 4, 5, 7, 8, PC3, 5, 6, 7, LR3, 14, GV11, 20, 24, BL14, 15, 18, 62, KI9, and the extra points *Si Shen Cong* (“four mind points”), *An Mian* (“peaceful sleep”), and *Yin Tang* (“hall of impression”) [49]. In the current paper, four out of eight trials reported mental functioning [20, 24–26] but only GV20 and LR3 were used in two trials [20, 24]. The rest of the trials did not use any points aiming to calm the mind. This discrepancy in point selection might have explained a lack of any effect on mental functioning at immediate and short-term followups. It is interesting to note that the real acupuncture had a delayed, small effect on improving mental function. To what degree this is related to the long-term effect on the physical functioning is unknown. As shown in Figure 3, it is possible that the elements of QoL improvement depend on the point selection. Future acupuncture trials in pain need to pay special attention to point selection to address both pain and mental functioning. 

The STRICTA data also indicated variation in the training and clinical experience of trial acupuncturists. The duration of training ranges from 140 hours to 4 years. Trial acupuncturists' experience ranges from no mention of experience to more than 15 years. Acupuncturists require a sufficient knowledge and skill base as well as clinical experience to enable them to provide successive acupuncture treatments of a consistent quality. Such consistency is an important aspect in the reproducibility and validity of trials. The recent CONSORT statement on nonpharmacological interventions stated that formally trained and skilled practitioners are essential for the success of non-pharmacological interventions [50]. To what degree a discrepancy in training might have impact on the trial results is unknown, and this requires further systematic investigation.

## 5. Conclusion

For patients with PAWS, there is moderate evidence suggesting that acupuncture may improve physical functioning and pain more effectively than those in the waiting-list or SI groups. Acupuncture may have a delayed, small effect on mental functioning. Acupuncture points used to improve mental functioning should be taken into consideration in future clinical trials for pain. A consensus needs to be reached concerning acupuncture treatment regimen and practitioners' training and clinical experience.

## Figures and Tables

**Table 1:** Demographics and outcome assessment.

Study	Randomisation method	Blinding	Sample size/dropout rate	Age (yr)	M/F	Multicentred trial? Acupuncturist versus participants	Time injury	Types of condition	Inclusion criteria	Outcome measure/followup/methods
Brinkhaus et al. [19]	Central	NM	301/19	40–75	96 : 202	Yes, 45 : 301	>6 mths	LBP	VAS> 40 out of 100 mm for 7 days before tx, only used NSAID in 4 wks before tx.	(i) A modified pain questionnaire (ii) pain disability index PDI (German) (iii) emotional aspects of pain (Schmerzempfindungsskala) (iv) depression scale (Allgemeine Depressionsskala) (v) SF-36 (German) (vi) Number of days with pain and taking pain medication /Followup for 52 wks/NM

Irnich et al. [20]	computer	AB	177/12	Age was not provided for the entire population. Age was only provided for each group (Acupuncture: 52.3 ± 13.3Massage: 52.7 ± 11.5Sham laser: 52.2 ± 13.2).	60 : 117	Yes, 4 : 117	>1 mth	NP	Painful restriction of neck mobility, no tx for 2 wks before.	(i) Change in the maximum pain related to motion (ii) Active ROM with a 3D ultrasound real time motion analyser (iii) VAS score of 6 cervical spine ROM (iv) pressure pain threshold bilaterally at levator scapulae, trapezius descendens, and paravertebral of C6. (v) SF-36 /3 mths/NM

Itoh et al. [21]	computer	PAB	26/5	≥65	9 : 17	no	≥6 mths	LBP	Normal neurological findings of lumbosacral nerve function, leg pain permitted if less than LBP.	(i) VAS (ii) RMQ /3 wks (But the followup data were not meta-analysed due to treatment group crossed)/NM.

Kerr et al. [22]	computer	AB	60/14	41 ± 12.6	28/32	no	>6 mths	LBP	No neurologic deficits.	(i) SF-36 (ii) Lumbar flexion ROM (iii) Pain rating index of MPQ and VAS /6 mths/postal questionnaire

Kennedy et al. [23]	computer	PAB	48/8	18–70	23/25	Unknown. 3 : 48	<12 wks	LBP	LBP with/without leg pain	(i) VAS (ii) RMQ (iii) a multidimensional patient-centred questionnaire /3 mths/NM

Vas et al. [24]	computer	AB	123/8	≥17	22/101	no	>3 mths	NP	Uncomplicated neck pain with motion related neck pain VAS ≥ 30 out of 100 mm.	(i) VAS (ii) Northwick Park Neck Pain Questionnaire (iii) SF-36 (Spanish) /6 mths/NM.
White et al. [25]	computer	PB	135/28	18–80	48/87	No	>2 mths	NP	VAS > 30 out of 100 mm for previous 5 out of 7 pre treatment days.	(i) VAS (ii) Neck Disability Index (iii) SF-36 (iv) Borkovec and Nau scale /52 wks/NM

Witt et al. [26]	Central	No blinding	2841/	≥18	1213/1628	Yes, 3486 : 11630	>6 mths	LBP	Chronic LBP	(i) standardized questionnaires (including sociodemographic characteristics) (ii) Hannover Functional Ability Questionnaire (iii) SF-36 /6 mths/questionnaire

AB: Assessor blinded;

Central: Randomised by central telephone randomisation procedure;

computer: Randomised by computer software

LBP: Low back pain;  MPQ: McGill Pain Questionnaire;      Mth: month;          NM: not mentioned

NP: Neck pain;     PAB: Patient and assessor blinded;       PB: Patient blinded;            PDI: Pain Disability Index

RMQ: Roland Morris Disability Questionnaire;             SF-36: Short-form 36 health survey;

tx: treatment;       VAS: Visual analogue scale; wk: week    Wk:week.

**Table 2:** A summary of meta-analysis results: acupuncture versus waiting list.

	Immediate followup	Short-term followup	Intermediate-term followup	Long-term followup
Physical functioning	Favours acupuncture, *n* = 1	Favours acupuncture, *n* = 1	*	*
Mental functioning	No difference, *n* = 1	Favours acupuncture, *n* = 1		
Pain	Favours acupuncture, *n* = 1	Favours acupuncture, *n* = 1		

*n* =  number of studies in this comparison.

*No data available for this comparison.

**Table 3:** A summary of meta-analysis results: acupuncture versus SI (Sham intervention).

	Immediate followup	Short-term followup	Intermediate-term followup	Long-term followup
Physical functioning	Favours acupuncture, *n* = 6	No difference, *n* = 4	Favours acupuncture, *n* = 2	*
Mental functioning	No difference, *n* = 3	No difference, *n* = 1	Favours acupuncture, *n* = 2	
Pain	Favours acupuncture, *n* = 7	Favours acupuncture, *n* = 3	No difference, *n* = 3	

*n* =  number of studies in this comparison.

*No data available for this comparison.

**Table:** (a)

Study	Rationale of acupuncture	Style of acupuncture (L versus D points, APs, TPs)/types of stimulation	U versus B/number of needles used	Needle retention time/treatment regiment (described in number of treatment/period)/De Qi.
Brinkhaus et al. [19]	NM	L and D, APs, TPs/M	B/> = 10	30 min/12 x/8 wks (2 x/wk for 4 wks followed by 1 x/wk for 4 wks.)/yes.

Irnich et al. [20]	TCM and other points.	L and D based on the affected meridians, APs and TPs/NM	NM/NM	30 min/5 x/3 wks/NM, local twitch is elicited for TPs.

Itoh et al. [21]	TP theory	TP/NM	NM/2–7	10 min/NM/NM, but stated to achieve “local twitch response”.

Kerr et al. [22]	CTs and TI	L and D/M	B/11	30 min/6 x/6 wks/yes.
Kennedy et al. [23]	TI, CTs, and expert's opinion.	L and D/M with even technique	U or B depending on patient's pain/8 to 13	30 min/3–12 x/4–6 wks/yes.

Vas et al. [24]	TI	L and D, APs/M	B/7 to 16	30 min/2 x/wk for 2 wks followed by 1 x/wk for 1 wk/yes.

White et al. [25]	TI and experts consensus.	L and D/M	B when pain is B/6 on average.	20 min/2 x/wk for 4 wks/yes

Witt et al. [26]	At the physicians' discretion.	At the physicians' discretion/At the physicians' discretion, but electroacupuncture, laser acupuncture and moxibustion were not permitted.	At the physicians' discretion/at the physicians' discretion.	At the physicians' discretion/15 x/3 mths/at the physicians' discretion.

**Table:** (b)

Author and date	Acupoints used in the trial	Acupuncturist's training	Cointervention
Brinkhaus et al. [19]	BL20 to 34; BL50 to 54; GB30; GV3, 4, 5 and 6; Huatuojiaji and Shiqizhuixia. SI3; BL40, 60 and 62; KI3 and 7; GB31, 34 and 41; LR3 and GV14 and 20. For patients experiencing local or pseudoradicular sensation, at least two local points were acupunctured. APs and TPs could be chosen individually.	> = 140 hours of training, >3 yrs experience.	NSAID.

Irnich et al. [20]	Frequently used point SI3, BL10, BL60, LR3, GB20, GB34, TE5. APs: cervical. TPs in trapezius (near GB20) and levator scapulae (near SI14).	4 experienced licensed medical acupuncturists; training not mentioned.	None (even no concomitant analgesics).

Itoh et al. [21]	TPs based on individual patients' response.	4 yrs of training and 7 yrs of clinical experience.	NM.

Kerr et al. [22]	BL23, BL25, GB30, BL40, KI3, and GV4.	A chartered physiotherapist trained in acupuncture; experience not mentioned.	A leaflet included standardized advice and exercise.

Kennedy et al. [23]	GV3, GV4, BL23, 25, 36, 37, 40, 56, 60, GB29-31, 34.	Senior experienced physiotherapists with > = 10 yr experience and were members of the Acupuncture Association of Chartered. Physiotherapists.	Staying active and routine medications.

Vas et al. [24]	GB20, GB21, LR3, LI4, GB34, BL10, GV14, SI3, BL62, GB39, Yintang, GV20, SP6. AP: shenmen, neck, liver, muscle relaxation, occiput, thalamus, ear kidney.	Accredited by the Beijing University of Medical Science (China) and >15 yrs clinical experience.	Auricular seeds and rescue medication (diclophenac) (if pain relief not obtained).

White et al. [25]	Unable to locate the list of points.	Trained with the Association of Chartered Physiotherapists and 7 yrs clinical experience.	Acetaminophen.

Witt et al. [26]	At physician's discretion.	At least > = 140 hours of training and wide variation trainings in style and acupuncture technique.	Conventional treatments.

AP: Auricular point; B: Bilateral; CT: controlled trials; D: Distal; L: Local.

LBP: Low back pain; M: Manual; Mth: month; NM: not mentioned.

TI: Textbooks information; TP: Trigger point; U: Unilateral.

Wk: week; TCM: traditional Chinese Medicine; yr: year.
